# Exploring the potential protective role of anthocyanins in mitigating micro/nanoplastic-induced reproductive toxicity: A steroid receptor perspective

**DOI:** 10.1016/j.jpha.2024.101148

**Published:** 2024-11-14

**Authors:** Jiaojiao Zhang, Wenyi Liu, Fuqiang Cui, Marjukka Kolehmainen, Jing Chen, Lei Zhang, Iman Zarei

**Affiliations:** a College of Food and Health, Zhejiang A&F University, Hangzhou, 311300, China; b College of Forestry and Biotechnology, Zhejiang A&F University, Hangzhou, 311300, China; c Institute of Public Health and Clinical Nutrition, School of Medicine, Faculty of Health Science, University of Eastern Finland, Kuopio, 70211, Finland; d Department of Chemical Engineering and Waterloo Institute for Nanotechnology, University of Waterloo, Waterloo, N2L3G1, Canada

**Keywords:** Microplastics and nanoplastics, Anthocyanin, Endocrine-disrupting chemicals, Reproductive toxicity, Steroid receptors, Environmental contaminants/pollutants

## Abstract

Microplastics and nanoplastics (MPs/NPs) are ubiquitous environmental pollutants that act as endocrine-disrupting chemicals (EDCs), raising significant concerns about their impact on human health. Research highlights the hazardous effects of MPs/NPs on both male and female reproductive systems, influencing germ cells, embryo development, and progeny. Additionally, studies show that MPs/NPs affect the gene expression of anabolic steroid hormones *in vitro* and *in vivo*, inducing reproductive toxicity through mechanisms such as oxidative stress and inflammation. Considering these adverse effects, identifying natural compounds that can mitigate the toxicity of MPs/NPs is increasingly important. Plants offer a wealth of antioxidants and anti-inflammatory compounds that can counteract these harmful effects. Among these, anthocyanins, natural colorants responsible for the vibrant hues of fruits and flowers, exhibit a wide range of biological activities, including antioxidant, anti-inflammatory, and anti-neoplastic properties. Moreover, anthocyanins can modulate sex hormone levels and alleviate reproductive toxicity. Cyanidin-3-glucoside (C3G), one of the most extensively studied anthocyanins, shows promise in reducing reproductive toxicity, particularly in females, and in protecting male reproductive organs, including the testis and epididymis. This protective effect is believed to result from its interaction with steroid receptors, specifically the androgen and estrogen receptors (ERs). These findings highlight the need to explore the mechanisms by which anthocyanins mitigate the reproductive toxicity caused by MPs/NPs. This review provides novel insights into how natural compounds can be leveraged to lessen the impact of environmental contaminants on human health, especially concerning reproductive health.

## Highlights

• PE, PS, PP, PET, and PVC degrade into MPs/NPs, accumulating and harming the reproductive system.
• MPs/NPs harm fertility and pregancy via oxidative stress, hormonal disruption, and inflammation.
• MPs/NPs impact reproductive health through pathways like p38 MAPK and Nrf2.
• Anthocyanins may support reproductive health by modulating estrogen receptors ERs.

## Graphical abstract

**Figure:**
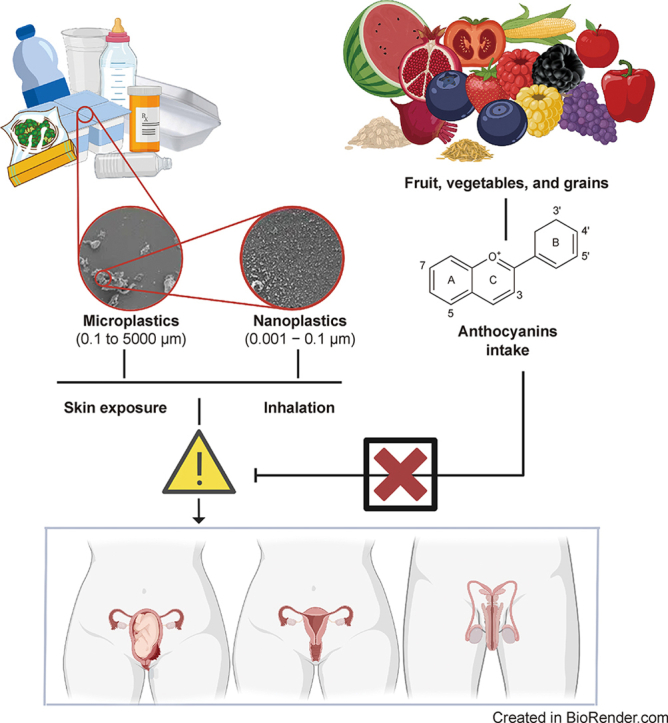

## 1 Introduction

Due to the versatility and enduring nature of plastics, these materials are found in widespread applications across various industries, particularly in the realm of food processes and packaging [1]. The accumulation of plastic waste contributes to plastic pollution in the terrestrial, marine, and atmospheric environments [2], where the most commonly found plastic polymers in food packaging include polyethylene (PE), polystyrene (PS), polypropylene (PP), PE terephthalate (PET), and polyvinyl chloride (PVC) [3]. These polymers can degrade into various sizes, including macroplastics, microplastics (MPs), and nanoplastics (NPs) through physical, chemical, and biological processes [4].

MPs and NPs can be transmitted through the food chain and gradually accumulate within organisms at different trophic levels. As humans, positioned at the top of this food chain, they may suffer the most significant adverse effects of MP toxicity. The accumulation of MPs/NPs contaminants across ecosystems raises significant concerns for human health due to their potential dispersion within the human body through respiratory, integumentary, and digestive systems [5]. For example, chronic exposure to relatively low doses of PE-MPs/NPs has been shown to cause adverse health effects, mainly on the reproductive and immune systems [6]. Liu et al. [7] conducted a study investigating the impact of PE-NPs exposure on reproduction, gonad development, germline apoptosis, and DNA damage in nematodes. Their findings revealed that the reproductive toxicity of PE-NPs exposure mediated by activation of nuclear hormone receptor 14 (NHR-14), a receptor in *Caenorhabditis elegans* that is analogous to some human nuclear receptors and DNA damage checkpoints, as well as estrogenic hormone receptors. The toxicity of PS-NPs can exacerbate damage to the reproductive system of male mice by perturbing their redox homeostasis [8]. Moreover, prolonged exposure to PS-MPs could lead to damage in the testes and ovaries, potentially inducing alterations in reproduction and fertility. Interestingly, female mice appear to be more susceptible to PS-MPs exposure than their male counterparts [9]. Although the direct correlation between PP/PET-MPs/NPs and their impact on reproductive system has not been firmly established [10], some studies have suggested that the accumulation of PP/PET-MPs within the bodies of filter-feeding organisms (such as *Daphnia similis* and *Mytilus galloprovincialis*) could lead to toxicity accumulation in aquatic environments [11,12]. PVC has been associated with a reduction in fertility potential in male rats. The effect is attributed to the diminished quality and quantity of androgen and sperm [13]. The impact of MPs and NPs on reproductive health remains incompletely understood. However, there is evidence suggesting that they could disrupt the endocrine system, exert influence on fertility, and impair embryonic development [14,15]. Therefore, addressing the reproductive toxicity caused by MPs has become an urgent issue in need of resolution. While some studies indicated cyanidin-3-glucoside (C3G), a type of anthocyanins, could mitigate toxicity induced by MPs/NPs [16,17], there remains a scarcity of reported studies on measures for protecting against this toxicity.

In recent years, food-borne natural products like anthocyanins have attracted more and more attention due to their advantages in low toxicity and side effects and multi-target effects. Anthocyanins belong to the flavonoid class of phenolic compounds that are water-soluble pigments that give plants their red, purple, or blue colors. They are widely distributed in fruits, vegetables, beans, cereals, and nuts [18], including their derived products such as juices and wines. Over 700 varieties of anthocyanins, characterized by 30 distinct core structures, have been identified [19]. There are 27 plant families and 72 genera that contain anthocyanins. Anthocyanins can be classified into two subclasses based on their chemical structure: anthocyanins and anthocyanidins. Anthocyanins are glycosides, which means they have one or more sugar molecules attached to them. Anthocyanidins are the aglycones, which means they are the sugar-free forms of anthocyanins. The fundamental structure of anthocyanidins is characterized by a 3,5,7-trihydroxy-2-phenylbenzopyrane skeleton (also known as the flavylium cation), which serves as the basic scaffold for anthocyanin [[20], [21], [22]]. Notably, the majority of naturally occurring anthocyanins are glycosylated, with common sugars binding to their C3, C5, and C7 positions. These sugars include glucose, galactose, xylose, arabinose, rutinose, and rhamnose [23]. There are six most common types of anthocyanidins, i.e., cyanidin, delphinidin, pelargonidin, peonidin, petunidin, and malvidin [24], differring in the number and position of hydroxyl and methoxyl groups on the benzene rings, which affect the color, stability, and bioavailability of the anthocyanins [25]. Table 1 [26,27] shows the molecular structure and color of each anthocyanidin, alongside the corresponding fruits, vegetables, and grains based on the anthocyanin’s aglycon.

**Table 1:** The structures of anthocyanidin with different colors under alongside the corresponding fruits, vegetables, and grains [26,27].

Anthocyanidin	Structure	Color	Fruits, vegetables, and grains
Cyanidin		Magenta	Acai berry (*Euterpe oleracea* Martius), berry (*Berberis lycium* Royle), bilberry (*Vaccinium myrtillus* L.), blackberry (*Rubus fruticosus* L.), blackcurrant (*Ribes nigrum* L.), blueberry *(Vaccinium corymbosum* L.), cranberry (*Vaccinium oxycoccos* L.), Dabai (*Canarium odonthophyllum* Miq.), Maqui berry (*Aristotelia chilensis* (Mol.) Stuntz), Nitratia (*Nitraria tangutorun* Bobr.), Oregon grape (*Mahonia aquifolium* (Pursh) Nutt), pomegranate (*Punica granatum* cv. Mollar de Elche), raspberry (*Rubus idaeus* L.), red grape (*Vitis vinifera* L.), black carrots (*Daucus carota* ssp. sativus var. atrorubens Alef.), black soybean (*Glycine max* (L.) Merrill), purple corn (*Zea mays* L.), purple sweet potato (*Ipomoea batatas* L.), red cabbage (*Brassica oleracea* L. var. capitata L.), and rice (*Oryza sativa* L. cv. Heugjinju)
Delphinidin		Purple	Acai berry (*Euterpe oleracea* Martius), berry (*Berberis lycium* Royle), bilberry (*Vaccinium myrtillus* L.), blackberry (*Rubus fruticosus* L.), blackcurrant (*Ribes nigrum* L.), blueberry (*Vaccinium corymbosum* L.), Dabai (*Canarium odonthophyllum* Miq.), Maqui berry (*Aristotelia chilensis* (Mol.) Stuntz), Nitratia (*Nitraria tangutorun* Bobr.), Oregon grape (*Mahonia aquifolium* (Pursh) Nutt), pomegranate (*Punica granatum* cv. Mollar de Elche), red grape (*Vitis vinifera* L.), black soybean (*Glycine max* (L.) Merrill), red cabbage (*Brassica oleracea* L. var. capitata L.), and transgenic purple tomato (*Solanum lycopersicum* L. cv. Del/Ros1)
Malvidin		Purple	Oregon grape (*Mahonia aquifolium* (Pursh) Nutt), red grape (*Vitis vinifera* L.), and transgenic purple tomato (*Solanum lycopersicum* L. cv. Del/Ros1)
Pelargonidin		Red	Berry (*Berberis lycium* Royle), blackberry (*Rubus fruticosus* L.), Nitratia (*Nitraria angutorun* Bobr.), Oregon grape (*Mahonia aquifolium* (Pursh) Nutt), pomegranate (*Punica granatum* cv. Mollar de Elche), purple corn (*Zea mays* L.), and purple sweet potato (*Ipomoea batatas* L.)
Peonidin		Magenta	Acai berry (*Euterpe oleracea* Martius), berry (*Berberis lycium* Royle), bilberry (*Vaccinium myrtillus* L.), blackberry (*Rubus fruticosus* L.), blueberry (*Vaccinium corymbosum* L.), cranberry (*Vaccinium oxycoccos* L.), Oregon grape (*Mahonia aquifolium* (Pursh) Nutt), red grape (*Vitis vinifera* L.), black soybean (*Glycine max* (L.) Merrill), purple corn (*Zea mays* L.), purple sweet potato (*Ipomoea batatas* L.), and rice (*Oryza sativa* L. cv. Heugjinju)
Petunidin		Purple	Bilberry (*Vaccinium myrtillus* L.), blueberry (*Vaccinium corymbosum* L.), red grape (*Vitis vinifera* L.), and transgenic purple tomato (*Solanum lycopersicum* L. cv. Del/Ros1)

Anthocyanins are substantially consumed in human diets. The average anthocyanin intake in different countries varies depending on the availability and consumption of anthocyanin-rich foods. For instance, in Australia, the daily anthocyanin intake is approximately 24.17 ± 0.32 mg/day per adult [28]. In the United States, the average daily intake is around 12.5 mg/day/person [29], while in Asia, it is 37 mg/day/person. In Europe, anthocyanin consumption varies widely from 19.8 mg/day/person in the Netherland to 64.9 mg/day/person in man, and 18.4 mg/day/person in Spain to 44.1 mg/day for women in Italy [30]. Wild berries are integral components of traditional Nordic diets, with two commonly consumed types in Finnish diets being Lingonberry (*Vaccinium vitis-idaea*) and Bilberry (*Vaccinium myrtillus*), both of which are significant sources of anthocyanins [31]. While specific data on the daily anthocyanin consumption of the Finnish population is not directly reported, the average daily berry consumption in Finland is 24 g/day for men and 35 g/day for women [32]. Given that lingonberries and bilberries are commonly consumed berries in Finland, the estimated daily anthocyanin consumption in Finland is approximately 12 mg/day for men and 17.5 mg/day for women. These vibrant pigments contribute not only to the visual appeal of our meals but also to their potential health benefits. Their recognized health benefits include antioxidant [33], cardiometabolic protection [33], neuroprotection [33], obesity management [34], various types of cancer prevention [35], and support for gut health [36], among others. Furthermore, although these pigmented compounds are not considered essential nutrients, there has been an evaluation of the recommended daily intake. Specifically, China recommends a daily consumption of 50 mg per individual to reduce oxidative stress levels, thus reducing the risks linked to cancer, metabolic syndrome, diabetes, degenerative diseases, and other pathologies [37].

**Fig. 1:**
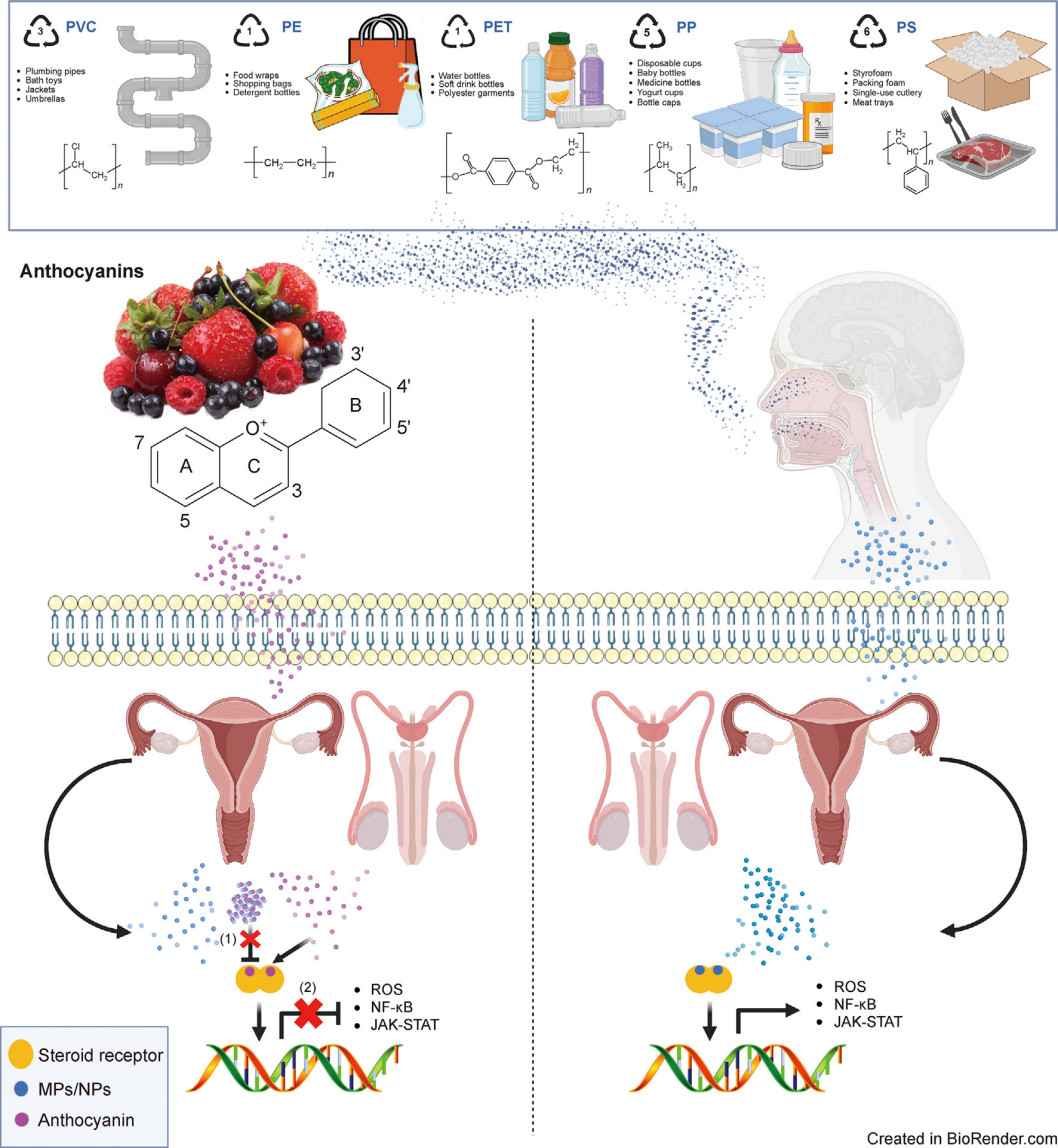
This steroid receptor-mediated protective role of anthocyanins against microplastics/nanoplastics (MPs/NPs)-induced reproductive toxicity. The intrusion of MPs/NPs into the biological environment can disrupt steroid receptor signaling, leading to altered gene expression that exacerbates oxidative stress and inflammation. MPs/NPs may trigger inflammation by activating inflammatory pathways such as nuclear factor κB (NF-κB) or cytokine-related signaling cascades like the Janus kinase (JAK)-signal transducer and activator of transcription (STAT) pathway, while also increasing oxidative stress by promoting the production of reactive oxygen species (ROS) in cells. Both inflammation and oxidative stress contribute to chronic inflammation, impaired function, and toxicity in reproductive tissues due to the deregulation of key pathways governed by steroid receptors. Anthocyanins may counteract these effects either by preventing MPs/NPs from interacting with steroid receptors (1) or directly interacting with steroid receptors (2) to downregulate the expression of genes involved in inflammation. PVC: polyvinyl chloride; PE: polyethylene; PET: PE terephthalate; PP: polypropylene; PS: polystyrene.

More specifically, anthocyanins are suggested to have potential in safeguarding against reproductive injury caused by oxidative stress, inflammation, or hormone imbalance in both males and females through the modulation of steroid receptors [38]. However, the underlying mechanisms by which anthocyanins influence signaling pathways remain unclear, requiring further investigation. Fig. 1 illustrates the proposed mechanism by which anthocyanins may exert their protective effects, potentially through the modulation of steroid receptors to mitigate MP/NP-induced reproductive toxicity. These complex mechanisms highlight the multifaceted role of anthocyanins in supporting reproductive health. Eisenreich et al. [39] investigated the effects of 3-chloro-1,2-propanediol (3-MCPD), a contaminant found in various processed foods in both its free form and as fatty acid esters, which is known to induce reproductive toxicity, particularly male antifertility effects. They studied the impact of 3-MCPD exposure and C3G intervention on male rats, and found that neither 3-MCPD exposure nor C3G intervention significantly affected the levels of luteinizing hormone (LH), testosterone, or dihydrotestosterone in the serum of the rats [40]. However, the C3G intervention did have a protective effect. It reversed the reduction in androgen receptor expression and safeguarded against damage to the testis, epididymis, and sperm caused by 3-MCPD exposure [39,41]. The above finding suggests a meaningful link between anthocyanins highlighting steroid hormones and intricate interactions between anthocyanins and hormonal pathways.

## 2 MPs/NPs and reproductive health

### 2.1 MPs/NPs and male reproductive health

Research indicates that exposure to MPs and NPs can negatively impact male reproductive health. A notable study has reported that an estimated minimum human equivalent dose for abnormal semen quality of 0.016 mg/kg/day [42]. Montano et al. [43] found that 6 of 10 semen samples contained 16 pigmented MP fragments. This study suggests that the presence of MPs in semen may indicate their traversal through the epididymis and seminal vesicles, potentially inciting inflammatory responses and adversely impacting male fertility. Zhao et al. [44] observed the presence of MPs with both testicular tissues and semen samples, with sizes predominantly falling within the 20–100 μm range. Intriguingly, the study revealed a higher average concentration of MPs in testicular samples as opposed to semen. Notably, PS was predominantly distributed in the testis, while PE and PVC were more prevalent in semen samples. A comparative study of humans and canines revealed significant disparities in the prevalence of certain MPs, such as acrylonitrile butadiene styrene (ABS) and PVC. This study found a significantly higher concentration of MPs in human testicular tissues, nearly three times that observed in canine counterparts [45]. Chen et al. [46] revealed that PS-NPs (25–100 nm) could infiltrate and impair human spermatozoa at concentrations as low as 5 and 50 μg/mL. Concurrently, PS-MPs (0.5−10 μm) were also found to adhere to the sperm surface. Furthermore, a synergistic toxicological interaction has been observed between PS-NPs and bisphenol A (BPA), exacerbating the detrimental effects on human sperm. Additionally, 24-h exposure to PS-MPs/NPs led to accumulation in mouse testicular tissue, resulting in decreased sperm quality and testosterone levels 28 days post-exposure [47].

### 2.2 MPs/NPs and female reproductive health

MPs/NPs can enter the bloodstream, breach the blood-testis or placental barrier and accumulate in the testicles, ovaries, and placenta [48,49]. *In vivo* studies identify MPs/NPs in ovarian granulosa cells and breach the blood-testis barrier [[50], [51], [52]]. Recent studies have shown that MPs/NPs can cause oxidative stress, inflammation, endocrine disruption, and apoptosis in the ovarian and uterine tissues. These effects can lead to reduced fertility, impaired embryo development, and uterine fibrosis [13,14]. The presence of toxic metals such as lead (Pb), which can accumulate in reproductive organs, may exacerbate these effects by interfering with hormonal balance and oocyte quality [41]. For example, one study found that MPs/NPs exacerbated ovarian and uterine toxicity when combined with Pb [53]. Moreover, the impact of MPs/NPs on reproductive health is evident in instances such as PS-MPs/NPs being rapidly absorbed into mouse blood within 30-min oral administration, resulting the penetration of the blood-testis barrier after 4 h of oral intake [50].

MPs/NPs are rapidly absorbed into the bloodstream, with PS MPs/NPs penetrating the blood-testis barrier within 4 h of oral intake and ovarian granulosa cells shortly thereafter. This results in reduced anti-Müllerian hormone (AMH) levels, oxidative stress, and uterine and ovarian fibrosis [54,55]. Studies have also shown that MPs/NPs can impact ovarian function, decrease follicle numbers, and reduce estradiol levels [16,17]. MPs/NPs can breach the placental barrier, with 50 nm PS-MPs/NPs detected in human placental tissue [48,56]. Additionally, MPs were found to accumulate in multiple tissues, especially in the fetus, placenta, kidney, spleen, lung, and heart, and led to a variety of harmful effects on both fetal development and maternal health [57]. This breach influences normal embryo development and can adversely affect pregnancy outcomes [58]. For instance, exposure to 10 nm PS-MPs/NPs in pregnant mice increased embryo absorption rate and decreased the number and diameter of uterine arterioles, shifting cytokine secretion towards an immunosuppressive state [59]. This suggests that MPs/NPs could reduce uterine blood supply and negatively affect pregnancy outcomes due to immune disturbance. Currently, the primary toxicity mechanisms of MPs/NPs on embryos include oxidative stress injury, abnormal energy metabolism, and immune disturbance [60]. These collective investigations assert the impact of MPs/NPs on both male and female reproductive systems, influencing germ cells, embryo development, and offspring. Despite the advancement of related medical research to the mammalian level [[60], [61], [62]], it remains primarily reliant on rodent experiments. Therefore, it is imperative to establish a more reliable theoretical basis for future human studies.

## 3 Mechanism underlying the adverse impact of MPs/NPs on the reproductive system

Inflammatory and oxidative damage are two major sources of toxicity, as evidenced by numerous contemporary investigations [63,64]. Functioning as a signaling molecule, p38 mitogen-activated protein kinase (MAPK) responds to stress stimuli and orchestrates cellular reactions, including apoptosis and autophagy. This cascade emerges as a potential mediator of reproductive impairment in rodents. Specifically, inhibiting p38 MAPK has been shown to alleviate sperm injury induced by MPs/NPs, as well as mitigate the insufficiency of testosterone synthesis [65,66]. Another study revealed that the activation of the nuclear factor erythroid 2-related factor 2 (Nrf2)/heme oxygenase 1 (HO-1)/nuclear factor κB (NF-κB) signaling pathway, which is involved in cellular responses to oxidative stress and inflammation, is closely linked to the reproductive toxicity of MPs/NPs [67]. Additionally, Jin et al. [68] speculated that the alteration of male hormone levels by MPs/NPs might be connected to the LH-regulated LH receptor (LHR)/cyclic adenosine monophosphate (cAMP)/protein kinase A (PKA)/steroidogenic acute regulatory protein (StAR) pathway, whereas Zhou et al. [69] suggested that the deterioration of sperm quality and acrosome impairment might be associated with the suppression of autophagy genes triggered by MPs/NPs.

Furthermore, gut microbial imbalance might have a significant role in PS-MPs/NPs-induced reproductive toxicity by modulating the interleukin 17A (IL-17A) signaling pathway [70]. A study by Wang et al. [71] indicated that PS-MPs/NPs might trigger the death and self-destruction of testicular cells by stimulating the reactive oxygen species (ROS)/MAPK/hypoxia-inducible factor 1-alpha (HIF1α) signaling pathway. The toll-like receptor 4/nicotinamide adenine dinucleotide phosphate (NADPH) oxidase 2 (NOX2) signaling axis might be activated to cause oxidative damage, accompanied by stimulation of the Notch and transforming growth factor beta (TGF-β) signaling pathways, leading to uterine fibrosis [55]; initiating nucleotide-binding oligomerization domain (NOD), leucine-rich repeat (LRR) and pyrin domain-containing protein 3 (NLRP3)/caspase-1 [72] and protein kinase R (PKR)-like endoplasmic reticulum kinase (PERK)/eukaryotic initiation factor 2-alpha (eIF2α) [53] signaling pathways, causing the death of ovarian granulosa cells. Another study proposed that MPs/NPs-triggered disruption of cytoskeletons leads to cell death and damages ovarian granulosa cells. Recent studies indicate they can be harmful to female reproductive health [73]. The primary established mechanisms involve PS-MPs/NPs stimulating the wingless-related integration site (Wnt)/β-catenin signaling pathway, causing ovarian fibrosis [54]. Present embryo toxicity mechanisms encompass oxidative damage, impaired energy metabolism, and immune dysregulation [60].

## 4 Anthocyanins and reproductive health

### 4.1 Anthocyanins and male reproductive health

Research, mainly from animal studies, suggests that anthocyanins may improve sperm quality, including sperm count and motility, by reducing oxidative stress in the tests. For example, C3G has been shown to significantly enhance male reproductive health in a mouse model of ulcerative colitis. In this study, C3G supplementation improved sperm count, testis weight, and spermatogenesis by reducing inflammation and protecting the integrity of the blood-testis barrier. Mice treated with C3G exhibited higher sperm quality, including increased sperm count and motility, and reduced testicular damage, indicating that C3G could be a promising therapeutic supplement for mitigating reproductive damage caused by inflammatory conditions like ulcerative colitis [74].

A study by Hu et al. [75] investigated the protective effects of anthocyanins on Leydig cells, which are responsible for testosterone production. The study found that oxidative stress induced by 2,2′-azobis(2-amidinopropane)-dihydrochloride (AAPH) leads to a reduction in testosterone synthesis, mainly by damaging the mitochondria and suppressing the StAR, which is crucial for steroid hormone production. The researchers tested four anthocyanins, i.e., C3G, delphinidin-3-glucoside (Dp-3-G), pelargonidin-3-glucoside (Pg-3-G), and cyanidin-3,5-diglucoside (C-3,5-G), to evaluate their effects in reversing these damages. They found that all four anthocyanins reduced oxidative stress and helped restore testosterone production, with C-3,5-G showing the strongest effect on restoring testosterone production. The study also highlighted that the structure of anthocyanins significantly influences their antioxidative activity and their ability to regulate StAR. Molecular docking simulations revealed that anthocyanins interact with StAR in a structure-dependent manner, further supporting their role in mitigating testosterone deficiency caused by oxidative stress.

### 4.2 Anthocyanins and female reproductive health

Evidence from both *in vitro* and *in vivo* studies shows that polyphenols could influence fertility and sexual developments [76]. Yang et al. [77] found C3G exhibits the potential to ameliorate female reproductive toxicity by cadmium (Cd). This is attributed to its capacity to induce estrogen-like effects, counteracting the impact of Cd, which functions as a metalloestrogen by binding to the estrogen receptor (ER) [78]. Endocrine-disrupting chemicals (EDCs), exemplified by BPA and phthalate esters, are incorporated during plastic manufacturing. Similar to Cd, they function by binding to ER, instigating hormonal responses, notably affecting estrogen, androgen, and thyroid hormones [76]. Anthocyanins have been documented to engage with ER, influencing pathways such as peroxisome proliferator-activated receptor γ (PPARγ), NF-κB, and Nrf2, among others [79]. In their intervention with anthocyanins in cell lines like MCF-7 and Ishikawa, known for elevated ERα expression, Nanashima et al. [80] explicitly elucidated the interaction between anthocyanins and ERα. Furthermore, anthocyanins not only modulate the expression of downstream genes influenced by ERα but also exert an influence on the expression levels of ERα. Modulation of membrane ER linked to G-proteins can regulate the phospholipase C (PLC)/diacylglycerol (DAG)/inositol triphosphate (IP3) cascade, intracellular Ca^2+^ mobilization, activation of protein kinase C (PKC), and even activate the adenylate cyclase/PKA pathway [81]. Another study explored the impact of anthocyanin on ovarian tissue damage and oxidative stress in rats subjected to ovarian torsion and detorsion (TD) [82]. Ovarian torsion is a serious condition that impairs blood flow, leading to ischemia, while TD, the restoration of blood flow, can exacerbate damage by increasing oxidative stress. The results showed that the TD group experienced significant ovarian tissue damage, oxidative stress, and hormonal imbalances, including lower levels of estrogen, glutathione peroxidase (GPX), and superoxide dismutase (SOD), alongside increased levels of malondialdehyde (MDA), a marker of oxidative stress. However, rats treated with anthocyanin showed significant improvements. Anthocyanin reduced MDA levels, restored GPX and SOD activity, and normalized estrogen levels, mitigating the oxidative damage and preserving ovarian tissue, particularly by protecting against follicle degeneration, suggesting that anthocyanin is an effective treatment for reducing oxidative stress and tissue damage associated with ovarian torsion and TD. Its antioxidant properties help preserve ovarian function and potentially maintain fertility, highlighting its therapeutic potential in managing ovarian damage.

To better understand how anthocyanins mitigate the adverse effects of MPs/NPs, in relation to the androgen/ERs, molecular simulations were conducted [83]. These simulations examined the interactions between anthocyanins and androgen/ERs. The results showed that plastic monomers had stronger interactions with the androgen receptor compared to anthocyanins did, while the interactions with the ER showed the opposite trend [83].

ERs are particularly prevalent in reproductive systems of both in male and females. Estrogen binding to these receptors regulates the expression of target genes, which is crucial for reproductive health and function. ERα is primarily located in the uterus and breast [84], whereas ERβ is more broadly distributed, including in the ovaries and other parts of the female reproductive system. ERβ plays a role in processes such as embryo transport, implantation, and maintaining normal reproductive cycles in the female reproductive system [84,85].

Research also indicates that ERα and ERβ are present in testicular cells [[86], [87], [88]], including Leydig and Sertoli cells, which are involved in key functions such as spermatogenesis, fluid regulation in the epididymis, and overall fertility [[87], [88], [89]].

## 5 Conclusion

The pervasive presence of MPs/NPs in the environment has raised significant concerns about their impact on human health, particularly reproductive toxicity. Both *in vitro* and *in vivo* studies highlight the potential risks posed by MPs/NPs, which include oxidative stress, endocrine disruption, and inflammation, all of which can impair reproductive function in both males and females. The search for natural compounds to counteract these harmful effects is ongoing, with anthocyanins emerging as a promising candidate. Anthocyanins such as C3G have demonstrated potential protective effects against reproductive toxicity induced by MPs/NPs. These compounds may exert antioxidant and anti-inflammatory effects, mitigate oxidative damage, and improve the function of steroid receptors such as androgen and ERs, which are crucial in maintaining reproductive health. The modulation of these receptors by anthocyanins may help restore hormonal balance, reduce cellular stress, and protect reproductive organs from plastic-induced damage. Future research should focus on the molecular pathways through which anthocyanins interact with MPs/NPs and their associated reproductive toxicity. Understanding these mechanisms can offer valuable insights into the development of anthocyanin-based therapeutic interventions to protect human reproductive health from environmental contaminants.

## CRediT authorship contribution statement

**Jiaojiao Zhang:** Conceptualization, Data curation, Funding acquisition, Investigation, Methodology, Project administration, Writing − original draft, Writing – review & editing. **Wenyi Liu:** Investigation, Writing – review & editing. **Fuqiang Cui:** Resources. **Marjukka Kolehmainen:** Funding acquisition, Resources, Writing – review & editing. **Jing Chen:** Methodology. **Lei Zhang:** Writing − original draft, Writing – review & editing. **Iman Zarei:** Conceptualization, Data curation, Investigation, Methodology, Project administration, Writing − original draft, Writing – review & editing.

## Declaration of competing interest

The authors declare that there are no conflicts of interest.
